# Staying cool or staying safe in a human-dominated landscape: which is more relevant for brown bears?

**DOI:** 10.1007/s00442-017-3948-7

**Published:** 2017-09-08

**Authors:** Andrés Ordiz, Ole-Gunnar Støen, Miguel Delibes, Jon E. Swenson

**Affiliations:** 1 Faculty of Environmental Sciences and Natural Resource Management, Norwegian University of Life Sciences, Postbox 5003, NO-1432 Ås, Norway; 2 Norwegian Institute for Nature Research, NO-7485 Trondheim, Norway; 3 Department of Conservation Biology, Estación Biológica de Doñana, Consejo Superior de Investigaciones Científicas, E-41092 Seville, Spain

**Keywords:** Bears, Conservation, Human disturbance, Management, Warming climate

## Abstract

Pigeon et al. (2016) Staying cool in a changing landscape: the influence of maximum daily ambient temperature on grizzly bear habitat selection. Oecologia 181:1101. doi:10.1007/s00442-016-3630-5 analyzed the effect of ambient temperature on the habitat selection of grizzly bears (*Ursus arctos*) in Alberta, Canada. They concluded that temperature played a significant role in bear habitat selection and that it was unlikely that human activity introduced biases to the habitat selection of bears. However, Pigeon et al. did not consider variables related to human activities in their analyses. They also misinterpreted previous research that has accounted for temperature in the habitat selection of brown bears. There is much literature published on the negative effects of human disturbance on wildlife in general and on bears in particular. Downplaying the role of human disturbance could have important negative consequences if, in fact, human disturbance were a more important factor than thermoregulation. Indeed, dismissing the importance of human influence, in the face of contradictory evidence, could tempt managers to disregard an important factor that is difficult and often unpopular to deal with in their conservation plans.

It is well documented that human disturbance can affect the behavior and habitat use of wildlife (e.g., Harrington and Veitch 1992; Beale and Monaghan 2004; Müllner et al. 2004; Blanc et al. 2006; Moore and Seigel 2006; Tuomainen and Candolin 2011). Thus, management efforts to conserve threatened or endangered species often involve managing or restricting human activities (e.g., Mattson et al. 1996; Richardson and Miller 1997; Williams et al. 2013; Trouwborst 2015; Sutherland et al. 2015), which is often not popular among the affected people (Woodroffe et al. 2005; Redpath et al. 2013 for reviews on a variety of conservation conflicts).

Pigeon et al. (2016) analyzed the effect of ambient temperature on the habitat selection of grizzly bears (i.e., brown bears *Ursus arctos*) in a landscape with a heavy human footprint in Alberta, Canada. They acknowledged that bear habitat selection is mainly driven by food, intra-specific factors (sex and reproductive status), and avoidance of human activity, but concluded that temperature played a significant role in bear habitat selection and argued that in a changing climate, large mammals may increasingly need to adjust spatial and temporal selection patterns in response to thermal constraints. Although they did not consider variables related to human activities, they also concluded that it was unlikely that human activity introduced temporal, spatial, or sex-related biases to the habitat selection patterns that they observed for male and female grizzly bears.

We feel that it is important to reexamine the role of human disturbance in affecting the behavior and habitat use by brown bears. Pigeon et al. did cite studies carried out in Alberta that show that human development, activities, and human-caused mortality influence bear habitat selection (Gibeau et al. 2002; Nielsen et al. 2004, 2010). Generally, human disturbance has been documented to be a major factor influencing brown bear habitat selection across the widespread range of the species in the Holartic (Naves et al. 2003; Rode et al. 2006; Nellemann et al. 2007, among many others). It is important to examine the conclusions of Pigeon et al. because it would make the conservation of threatened and endangered bear populations much easier if factors other than human disturbance were most important in influencing the bears’ habitat use. Of course, downplaying the role of human disturbance could have important negative consequences if, in fact, human disturbance were a more important factor than thermoregulation.

Pigeon et al. (2016) measured temperature across habitat types and took into account foraging requirements of bears, sex, time of the day, and three periods during summer to control for daily and seasonal variation in bear activity. However, we see as a major weakness of their study that they did not include any variable to evaluate the effect of human activities or related infrastructures among the predictors that may explain bear habitat selection.

In support of their conclusions, Pigeon et al. stated that there are no studies specifically linking temperature to habitat selection patterns for terrestrial bear species. However, they cited McLellan and McLellan (2015), who actually documented that when bears were foraging on berries in an open landscape, there was no relation between daily maximum temperature (from 20.4 to 40.1 °C) and the total amount of time that the bears were active. In addition, McLellan and McLellan found no difference in bear activity levels during day or night between warm (20.4–27.3 °C) and hot (27.9–40.1 °C) days. Therefore, McLellan and McLellan concluded that food acquisition had a stronger influence on activity levels of grizzly bears than heat dissipation. We also accounted for ambient temperature in our paper on the selection of resting sites by brown bears in Sweden (Ordiz et al. 2011, also published in Oecologia). Pigeon et al. cited this paper and correctly summarized our major findings, that human activity was the most important factor we examined in influencing the use of resting sites by brown bears. However, Pigeon et al. wrongly stated that we did not consider the potential for temperature-mediated selection of denser cover during daytime and suggested that “thermoregulatory needs could have played a role in the selection for dense cover observed by Ordiz et al. (2011)”.

We did acknowledge that temperature might influence the selection of bed sites and, therefore, installed temperature loggers at six permanent sites in the main habitat types present in our study area. Temperature and daylight length were highly correlated (*r* = 0.87), thus we included only daylight length in the final selection process because it was a more explanatory variable in our analyses; a candidate model with temperature instead of daylight length and all other variables being equal, had an AIC larger (546.6) than the model with daylight length (540.9). Furthermore, daylight length was a relevant variable to include because bear hunting is allowed only during the day. In our study, daylight length, a surrogate of both temperature and the time of the day when hunting occurred, and the human-associated variables (distance to human settlements and day/night) influenced bear selection of cover at beds the most. Bears selected denser habitats, not just denser cover, at bed sites when human activity was more intense and dispersed.

Most importantly, our conclusion that human disturbance was more important than thermoregulation has been corroborated in recent years by studies showing that human activities affect brown bear behavior and demography at different scales in Scandinavia, where bear mortality is mostly human caused (Bischof et al. 2009; Steyaert et al. 2016). Human activities affect bear habitat selection at different temporal and spatial scales (Moe et al. 2007; Nellemann et al. 2007; Martin et al. 2010), and bears become less diurnal after bear hunting seasons start (Ordiz et al. 2012) and where road density is higher (Ordiz et al. 2014, 2017). The conclusion that bears seek concealment cover to avoid humans (Ordiz et al. 2011) is further supported by the finding that, after experimental approaches to collared bears, they move to places even more concealed (Salhén et al. 2015). Wolves have shown the same result after approaches (Wam and Hjeljord 2012). After encounters with people, bears were also less active during daytime and more active during nighttime for a number of days (Ordiz et al. 2013).

As Pigeon et al. (2016) noted in their introduction, thermoregulatory needs are important for wildlife in general and it is intuitive to expect that temperature can also play a role in bear habitat selection. Nevertheless, there is nowadays a quite convincing bulk of literature documenting the effects of human activities on wildlife in general (e.g., Boyle and Samson 1985; Frid and Dill 2002; Blanc et al. 2006 for non-consumptive effects of human activities), and bears in particular. Humans cause most mortality in virtually all large carnivore populations (Woodroffe and Ginsberg 1998; Treves 2009), including bears (e.g., Sánchez-Mercado et al. 2008; Bischof et al. 2009). Human activities cause habitat fragmentation and habitat loss for different bear species (Liu et al. 1999; Naves et al. 2003; Escobar et al. 2015; Puri et al. 2015; Andersen and Aars 2016). Bears perceive changes in the level of risk posed by human activities, which can trigger behavioral responses (e.g., Stillfried et al. 2015) and/or stress responses (Støen et al. 2015; Ditmer et al. 2015). Not surprisingly, human activities are a core issue when planning conservation actions, e.g., to increase landscape connectivity (Brodie et al. 2015) and to establish management areas to reduce disturbance to, and habituation by, bears (Coleman et al. 2013).

Brown bears in Alberta are no exception, with many studies reporting severe effects of human activities on bear behavior and demography (e.g., Gibeau et al. 2002; Mueller et al. 2004; Munro et al. 2006; Nielsen et al. 2004, 2010; Ross 2002; COSEWIC 2012; Bourbonnais et al. 2013; Linke et al. 2013, among many others). For instance, present low bear densities and distribution in Alberta are associated with human disturbance (Linke et al. 2013), and >80% of documented bear mortality is caused by people (Bourbonnais et al. 2013).

Whereas we do not cast doubt on the conclusion of Pigeon et al. (2016) that large mammals, including bears, may increasingly need to adjust spatial and temporal selection patterns in response to changing thermal constraints (but see McLellan and McLellan 2015), we suggest that human disturbance should have been taken into account quantitatively in their study. Otherwise, the statements on the lack of human influence on bear habitat selection are not supported by any data, and their results may have been different if human factors had been included. Our criticism of Pigeon et al.’s (2016) conclusion that human influence was not important for habitat selection of the threatened brown bear in Alberta is not trivial. Failing to include factors of human influence into their analysis and dismissing the importance of human influence, in the face of so much contradictory evidence, could tempt managers (in Canada and elsewhere) to disregard an important factor that is difficult and often unpopular to deal with in their conservation plans.
